# Supplementary material for: Simulating anti-skyrmions on a lattice
Source: Sci Rep. 2022 Nov 10;12:19179. doi: 10.1038/s41598-022-22043-0 (PMC9649801; doi:10.1038/s41598-022-22043-0)
Supplement: Supplementary file 1 — Supplementary Information. [file 41598_2022_22043_MOESM1_ESM.pdf]

Supplementary information: Simulating anti-skyrmions on a lattice

Juan C. Criado[1], Sebastian Schenk[1], Michael Spannowsky [1], Peter D. Hatton[2], L. A. Turnbull[2]

[1]*Institute for Particle Physics Phenomenology, Department of Physics, Durham University, South Road, Durham DH1 3LE, United Kingdom*

[2]*Centre for Materials Physics, Department of Physics, Durham University, South Road, Durham DH1 3LE, United Kingdom*

Modifying the DM interaction strength

In the main text, we have demonstrated that magnetic anti-skyrmion tubes are stabilized in a large region of parameter space. For this simulation we have fixed the (lattice) DM interaction coefficient to $\hat{K} = \tan(2\pi/10)$. Let us now explore the anti-skyrmion stability with respect to changes of this parameter. As we have seen that anti-skyrmions are consistently formed at a temperature of about $\hat{T} = 0.9$, we keep the latter value fixed to study deformations with respect to the DM interaction strength.

In Fig. S1, we show the corresponding phase diagram for $\hat{B}$ and $\hat{K}$, using an experimentally motivated ZFC schedule. As an additional motivation for the use of this schedule, we notice that, among the phase diagrams in Fig. 3 of the main text, the one with ZCF schedule has the largest range of values of $\hat{B}$ in which anti-skyrmion phase appears. This schedule thus becomes

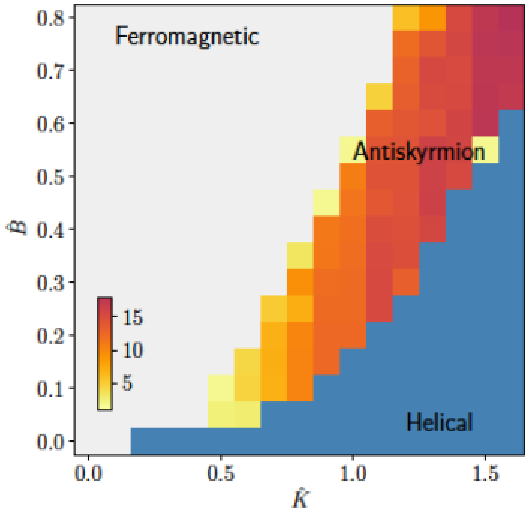

Figure S1: | **Anti-skyrmion lattice phase within the DM interaction coefficient $\hat{K}$ and the magnetic field $\hat{B}$.** Here we use a ZFC schedule to a fixed target temperature of $\hat{T} = 0.9$ everywhere. The color-coding illustrates the total antiskyrmion number, i.e. the antiskyrmion phase is shown in red. At low $\hat{K}$, a ferromagnetic phase forms because the effects of the ferromagnetic exchange interactions dominate. In this phase, the spins point in the positive z direction, except at $\hat{B} = 0$, where no specific direction is preferred.

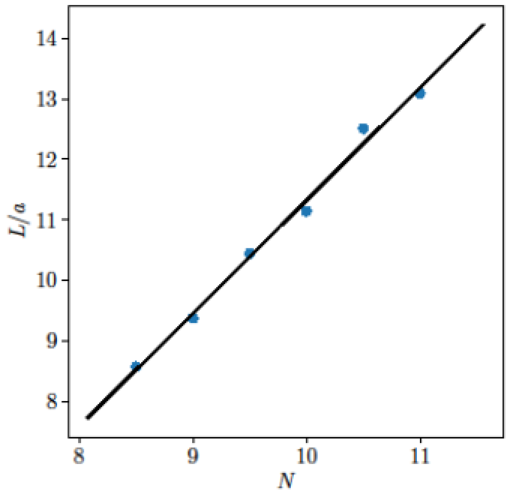

Figure S2: | **Average anti-skyrmion size in units of the lattice spacing, L/a, as a function of the helical period in units of the lattice sites, N, (cf. main text Eq. 13).** Here, we consider a target temperature and magnetic field of $\hat{T} = 0.9$ and $\hat{B} = 0.2$, respectively. These are obtained by performing a ZFC schedule on a $60 \times 60 \times 30$ spin lattice

the most adecuate for generating Fig. S1, which we will use for the determination of the largest region in parameter space in which this phase exists. We observe that larger values of $\hat{K}$ require a larger magnetic field in order for anti-skyrmion tubes to form. Since materials with a large DM parameter are rare in practice, we note that experiments might look for anti-skyrmions in materials with reasonably low DM interaction strength using a comparably small magnetic field. However, at the same time, this approach is limited by the fact that, if the magnetic field is too small, the anti-skyrmion lattice phase disappears completely. Physically, in this case, the positive energy contribution by the magnetic field interaction is too small, thereby failing to stabilize the solitons. This may set a lower limit on the DM interaction strength to stabilize anti-skyrmion tubes. Extrapolating the approximately linear behaviour of the minimal $\hat{K}$ as a function of $\hat{B}$ to the value $\hat{B} = 0$, we obtain a bound $\hat{K} \gtrsim 0.3$. While a more detailed study with a finer scan in the free parameters (and the use of different schedules to ensure that the ground state is found) would be in order to determine this limit more precisely, we take the results presented in Fig. S1 to be a strong indication for the estimate $\hat{K} \gtrsim 0.4$. Indeed, this value can be crucial in the choice of material in an experimental search for anti-skyrmions in chiral magnets.

In addition, intuitively, the DM interaction strength controls the typical size of spin structures that emerge on the lattice. Let us briefly quantify this explicitly, by defining the average anti-skyrmion size as

$$L \simeq \sqrt{\frac{A_{xy}}{Q_d}}\,. \tag{1}$$

Here, A_{xy} is the surface area of the lattice in the xy-plane and Q_d is the total topological charge given in main text (4). L thus the inverse square root of the anti-skyrmion number area density.

We count how many anti-skyrmion tubes can be densely packed into any given lattice volume. At the same time, in the helical phase, the period of the spin configurations (in terms of lattice sites) is given by [1]

$$N = \frac{2\pi}{\arctan \hat{K}} \,. \tag{2}$$

This is the defining estimate for the DM interaction coefficient that we used throughout this work for $N = 10$. Intrinsically, the average anti-skyrmion size should be related to the period of spin configurations. Indeed, in SI Fig. 1, we show the average anti-skyrmion size as a function of N, for constant temperature and magnetic field, $\hat{T} = 0.9$ and $\hat{B} = 0.2$, respectively. In this simulation, we use a $60 \times 60 \times 30$ lattice to reduce the effects of a finite lattice size and apply a ZFC annealing schedule. We find that, to good agreement, both quantities are related linearly,

$$\frac{L}{a} \approx 1.87N - 7.38 \,, \tag{3}$$

thereby supporting the fact that the DM interaction coefficient $\hat{K}$ is controlling the size of the anti-skyrmion spin textures. Here, again, a denotes the lattice spacing. Finally, we hope that this analysis can guide the experimental search for anti-skyrmions by identifying suitable magnetic materials. We note there have been some claims of anti-skyrmions existing in both chiral and centrosymmetric materials. However to date the objects observed do not seem to correspond to the anti-skyrmions stabilized by DM interactions observed by us in this study. We hope that the sizes and shapes and behavior we have found in this study may help to resolve potential ambiguities. However it is also noted that we are using a restricted model Hamiltonian and our study cannot produce magnetic objects such as magnetic bubbles that rely on dipole interactions, which may have been observed in previous experimental studies.

1. Yi, S. D., Onoda, S., Nagaosa, N. & Han, J. H. Skyrmions and anomalous Hall effect in a Dzyaloshinskii-Moriya spiral magnet. *Phys. Rev. B* **80**, 054416 (2009).
